# Anthelmintic Activities of Aporphine from *Nelumbo nucifera* Gaertn. cv. *Rosa-plena* against *Hymenolepis nana*

**DOI:** 10.3390/ijms15033624

**Published:** 2014-02-27

**Authors:** Rong-Jyh Lin, Mei-Hsuan Wu, Yi-Hsuan Ma, Li-Yu Chung, Chung-Yi Chen, Chuan-Min Yen

**Affiliations:** 1 Department of Parasitology, School of Medicine, College of Medicine, Kaohsiung Medical University, Kaohsiung 807, Taiwan; E-Mails: rjlin@kmu.edu.tw (R.-J.L.); u102800001@cc.kmu.edu.tw (Y.-H.M.); leyich@kmu.edu.tw (L.-Y.C.); 2 Department of Medicinal and Applied Chemistry, College of Life Science, Kaohsiung Medical University, Kaohsiung 807, Taiwan; E-Mail: u97021112@cc.kmu.edu.tw; 3 Department of Medical Laboratory Science and Biotechnology, School of Medical and Health Sciences, Fooyin University, Ta-Liao District, Kaohsiung 83102, Taiwan

**Keywords:** *Nelumbo nucifera* Gaertn, aporphine, *Hymenolepis nana*, *Anisakis simplex*, anthelmintic activity, peroxyl radical

## Abstract

*Nelumbo nucifera* Gaertn. cv. *Rosa-plena* (*Nelumbonaceae*), commonly known as lotus, is a perennial aquatic plant grown and consumed throughout Asia. All parts of *N. nucifera* have been used for various medicinal purposes in oriental medicine. From the leaves of *Nelumbo nucifera* Gaertn. cv. *Rosa-plena* (an aquatic plant), liriodenine (**1**), lysicamine (**2**), (−)-anonaine (**3**), (-)-asimilobine (**4**), (-)-caaverine (**5**), (-)-*N*-methylasimilobine (**6**), (-)-nuciferine (**7**), (-)-nornuciferine (**8**), (-)-roemerine (**9**), 7-hydroxydehydronuciferine (**10**) and cepharadione B (**11**) were isolated and identification and anthelmintic activities of aporphine was evaluated against *Anisakis simplex* and *Hymenolepis nana*. This study found that the above constituents killed *H. nana* or reduced their spontaneous movements (oscillation/peristalsis). However, the above constituents at various concentrations demonstrated no larvicidal effect or ability to halt spontaneous parasite movement for 72 h against *A. simplex*, respectively. In addition, according to an assay of cestocidal activity against *H. nana* and nematocidal activity against *A. simplex*, we found that the above compounds showed greater lethal efficacy on *H. nana* than against *A. simplex.* Further investigation showed that these above constituents have effects against peroxyl radicals under cestocidal effect. Together, these findings suggest that these constituents of *Nelumbo nucifera* Gaertn. cv. *Rosa-plena* might be used as anthelmintic agents against *H. nana.*

## 1. Introduction

**Figure 1.**
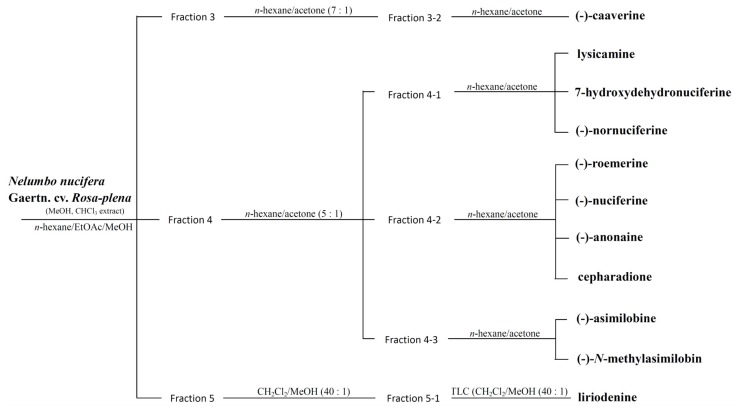
Schematic drawing of extraction from *Nelumbo nucifera* Gaertn. cv. *Rosa-plena* leaves.

**Figure 2.**
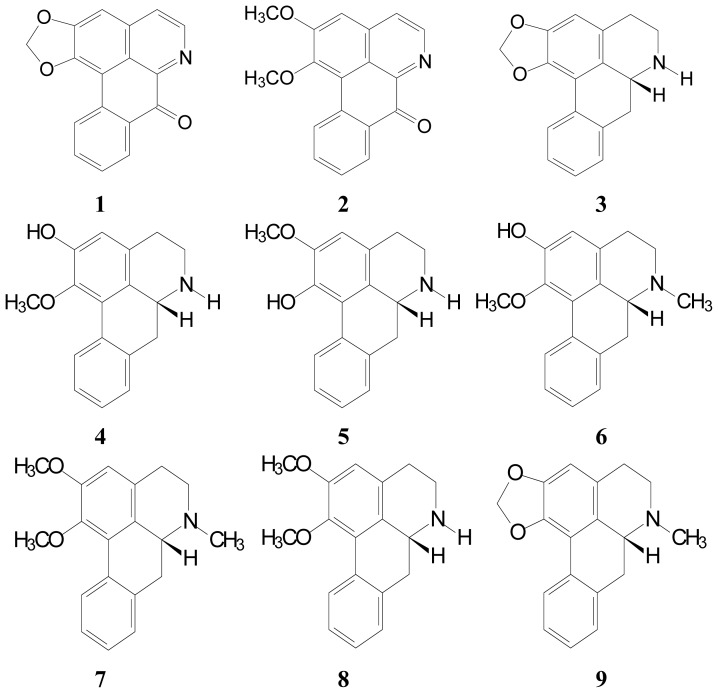

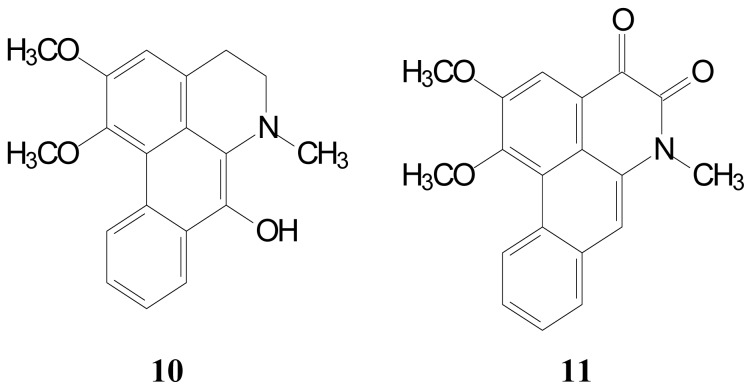
Structures of alkaloids (**1**–**11**) isolated from the leaves of *N. nucifera*.

*Nelumbo nucifera* Gaertn. cv. *Rosa-plena* is a perennial aquatic crop grown and widely distributed throughout Asia, and long used for food and medicine. All segments of *N. nucifera* have been used for various medicinal purposes in traditional oriental medicine. The matured leaf is fibrous and usually used as a health food in Asia. The leaves are famous for their astringent properties and use as diuretics, and are also used to treat sweating, fever, strangury and as a styptic [1]. To further understand the chemotaxonomy of the *Nelumbo* species [1], *Nelumbo nucifera* Gaertn. cv. *Rosa-plena* was chosen for phytochemical investigation and evaluated for bioactive compounds (Figure 1). We are not aware of any publications concerning the chemical components of this plant against parasites such as *Anisakis simplex* and *Hymenolepis nana*. The compounds derived from the leaves of *Nelumbo nucifera* Gaertn. cv. *Rosa-plena* include eleven aporphines: liriodenine (**1**), lysicamine (**2**), (-)-anonaine (**3**), (-)-asimilobine (**4**), (-)-caaverine (**5**), (-)-*N*-methylasimilobine (**6**), (-)-nuciferine (**7**), (-)-nornuciferine (**8**), (-)-roemerine (**9**), 7-hydroxydehydronuciferine (**10**) and cepharadione B (**11**) [1–3] (Figure 2).

*Hymenolepis nana* is a common opportunitistic cestode parasite and is found worldwide. *H. nana* infections are typically asymptomatic but heavy infections also cause anorexia, headaches, weakness, diarrhea, and abdominal pain [4]. *H. nana* infection is more dangerous for small children than adults, especially in regions with inadequate sanitation and hygiene. *H. nana* is the only cestode without any intermediate hosts in its life cycle [5]. *H. nana* infection is typically acquired from eggs in contaminated food. Eggs are ingested by an arthropod intermediate host and hatch in the duodenum, releasing oncospheres, and develop into cysticercoid larvae. Upon rupture of the villus, the cysticercoids return to the intestinal lumen, evaginate their scoleces, attach to the intestinal mucosa, and mature into adults that reside in the ileal portion of the small intestine, producing gravid proglottids. The eggs are then passed in stools when released from the proglottids or disintegration of proglottids in the small intestine. An alternate mode of infection consists of internal autoinfection without passing through the external environment. The short life span and rapid course of development also facilitates the spread and ready availability of this worm, but internal autoinfection allows the infection to continue for years [5].

*Anisakis nematodes* are marine mammal parasites and have fish and crustaceans as intermediate hosts. Anisakiasis is a widely distributed zoonosis associated with fish consumption. In humans they carry out an incomplete cycle as the larvae do not have the adequate environment to reach the adult stage [6]. Humans act as accidental hosts by consuming undercooked and/or raw second intermediate hosts that contain *A. simplex* third-stage larvae (AsL3). *A. simplex* rarely develops further within the human gastrointestinal tract, instead, by means of proteolytic enzymes, they typically become embedded in the gastric or intestinal mucosa and die, or invade the muscular layers of the stomach and intestine to induce allergic reactions and a variety of abdominal symptoms that are characterized as anisakiasis or anisakidosis [7]. Infection by the *A. simplex* [8–10] depends on both the viability of the larvae and on the activity of their somatic and/or secretory/excretory antigens. Four major clinical symptoms in human anisakiasis are gastric, intestinal, ectopic (extra-gastrointestinal) and allergic disease. Anisakidosis is globally recognized as a public health problem. It is relative to Asia and Europe [7]. The prevalence of anisakidosis has increased unusually because of the increasing popularity of Japanese cuisine, such as sushi and sashimi. The availability of an anthelmintic compound against *A. simplex* has the potential to shorten the clinical course and prevent invasive intervention from endoscopic procedures.

Free radical scavenging activities have been implicated in some inflammatory diseases. Some agents also have free radical scavenging activity and antiprotozoan activity [11]. However, free radical scavenging activity failed to reduce their larvicidal activity [12]. Other studies have suggested that free radical scavenging may reduce larvicidal activity by permitting larvae survival. Therefore, exactly how free radical scavenging affects the cestocidal activity of certain anthelminthic agents still remains unclear. Whether or not a correlation exists between the possible scavenger activity of aporphine and their anthelminthic activity is determined using oxygen radical absorbance capacity (ORAC) assays.

However, the bioactive mechanisms underlying cestocidal effects on *H. nana* and larvicidal effects on *A. simplex* of liriodenine (**1**), lysicamine (**2**), (-)-anonaine (**3**), (-)-asimilobine (**4**), (-)-caaverine (**5**), (-)-*N*-methylasimilobine (**6**), (-)-nuciferine (**7**), (-)-nornuciferine (**8**), (-)-roemerine (**9**), 7-hydroxydehydronuciferine (**10**) and cepharadione B (**11**) remain unclear. This study confirmed the anthelmintic activities against *H. nana* and *A. simplex* of above compounds. The objective of the present study was to investigate the possible anthelmintic activity against *H. nana* and *A. simplex* of different components of *Nelumbo nucifera* Gaertn. cv. *Rosa-plena* related to aporphine, exploring the possible use of these compounds as food additives for prophylaxis against parasite infection.

## 2. Results and Discussion

### 2.1. Isolation and Characterization of Aporphine Derivatives

**Liriodenine** (**1**) as in [13], yellow needles (CHCl_3_); UV λ_max_: 256, 280, 334 nm; IR ν_max_: 950, 1050, 1650 cm^−1; 1^H NMR (400 MHz, CDCl_3_): δ 6.37 (2H, s, –OCH_2_O–), 7.16 (1H, s, H-3), 7.55 (1H, td, *J* = 8.2, 1.6 Hz, H-9), 7.74 (1H, td, *J* = 8.2, 1.6 Hz, H-10), 7.76 (1H, d, *J* = 5.4 Hz, H-4), 8.61 (1H, dd, *J* = 8.2, 1.6 Hz, H-8), 8.64 (1H, dd, *J* = 8.2, 1.6 Hz, H-11), 8.89 (1H, d, *J* = 5.4 Hz, H-5); ESI-MS *m*/*z*: 275 [M]^+^ (Figure 2).

**Lysicamine** (**2**) as in [13], yellow needles (CHCl_3_); UV λ_max_: 255, 283, 335 nm; IR ν_max_: 1650 cm^−1; 1^H NMR (400 MHz, CDCl_3_): δ 4.04 (3H, s, C_1_–OCH_3_), 4.13 (3H, s, C_2_–OCH_3_), 7.21 (1H, s, H-3), 7.60 (1H, t, *J* = 6.0 Hz, H-9), 7.79 (1H, t, *J* = 6.4 Hz, H-10), 7.84 (1H, d, H-4), 8.61 (1H, d, *J* = 6.4 Hz, H-8), 8.95 (1H, d, H-5), 9.21 (1H, d, *J* = 6.4 Hz, H-11); ESI-MS *m*/*z*: 291 [M]^+^ (Figure 2).

**(-)-Anonaine** (**3**) as in [13], pale yellow powder (MeOH); UV λ_max_: 230, 272, 310 nm; IR ν_max_: 950, 1040 cm^−1; 1^H NMR (400 MHz, CDCl_3_): δ 2.65 (1H, t, *J* = 13.4 Hz, H-7a), 2.85 (1H, dd, *J* = 13.4, 5.2 Hz, H-7b), 3.11–3.29 (3H, m, H-4a, 4b, 5a), 3.53 (1H, m, H-5b), 3.98 (1H, dd, *J* = 13.4, 5.2 Hz, H-6a), 5.92 and 6.06 (each 1H, d, *J* = 1.6 Hz, –OCH_2_O–), 6.55 (1H, s, H-3), 7.21–7.30 (3H, m, H-8, 9, 10), 8.06 (1H, d, *J* = 7.6 Hz, H-11); ESI-MS *m*/*z*: 265 [M]^+^ (Figure 2).

**(-)-Asimilobine** (**4**) as in [14], brown powder (MeOH); UV λ_max_: 274, 308 nm; IR ν_max_: 3500 cm^−1; 1^H NMR (400 MHz, CDCl_3_) : δ 2.70–3.10 (4H, m, H-4, 5), 3.57 (3H, s, C_2_–OCH_3_), 6.70 (1H, s, H-3), 7.24–7.28 (3H, *m*, H-8, 9, 10), 8.26 (1H, *d*, *J* = 7.6 Hz, H-11); ESI-MS *m*/*z* : 267 [M]^+^ (Figure 2).

**(-)-Caaverine** (**5**) as in [15], brown powder (MeOH); UV λ_max_: 231, 271, 310 nm; IR ν_max_: 1625, 1760 cm^−1; 1^H NMR (400 MHz, CDCl_3_): δ 2.57–3.19 (4H, m, H-4, 5), 3.90 (3H, s, C_2_–OCH_3_), 6.66 (1H, s, H-3), 7.24–7.27 (3H, m, H-8, 9, 10), 8.37 (1H, d, *J* = 8.0 Hz, H-11); ESI-MS *m*/*z*: 267 [M]^+^ (Figure 2).

**(-)-*N*-methylasimilobine** (**6**) as in [14], white needles (CHCl_3_); UV λ_max_: 230, 272, 315 nm; IR ν_max_: 3600 cm^−1; 1^H NMR (400 MHz, CDCl_3_): δ 2.54 (1H, m, H-7b), 2.55 (3H, s, N-CH_3_), 2.62 (1H, m, H-4b), 2.65 (1H, m, H-5b), 2.69 (1H, m, H-7a), 3.04 (1H, m, H-5a), 3.12 (1H, m, H-6a), 3.14 (1H, m, H-4a), 3.58 (3H, s, C_1_–OCH_3_), 6.70 (1H, s, H-3), 7.24–7.28 (3H, m, H-8, 9, 10), 8.27 (1H, dd, *J* = 6.4 Hz, H-11); ESI-MS *m*/*z*: 281 [M]^+^ (Figure 2).

**(-)-Nuciferine** (**7**) as in [3], brown power (MeOH); UV λ_max_: 230, 274, 312 nm; IR ν_max_: 1250, 1375, 1425, 1500, 1605 cm^−1^, ^1^H NMR (400 MHz, CDCl_3_): δ 2.71–3.15 (4H, m, H-4, 5), 3.65 (3H, s, C_1_–OCH_3_), 3.88 (3H, s, C_2_–OCH_3_), 6.63 (1H, s, H-3), 7.22–7.26 (3H, m, H-8, 9, 10), 8.36 (1H, d, *J* = 7.6 Hz, H-11); ESI-MS *m*/*z*: 295 [M]^+^ (Figure 2).

**(-)-Nornuciferine** (**8**) as in [13], brown power (MeOH); UV λ_max_: 230, 272, 310 nm; IR ν_max_: 1440, 1590, 2900 cm^−1: 1^H NMR (400 MHz, CDCl_3_): δ 2.76–3.14 (4H, m, H-4,5), 3.65 (3H, s, C_1_–OCH_3_), 3.89 (3H, s, C_2_–OCH_3_), 6.64 (1H, s, H-3), 7.22–7.30 (3H, m, H-8, 9, 10), 8.36 (1H, d, *J* = 7.6 Hz, H-11); ESI-MS *m*/*z*: 281 [M]^+^ (Figure 2).

**(-)-Roemerine** (**9**) as in [3], pale brown powder (MeOH); UV λ_max_: 234, 272, 312 nm; IR ν_max_: 942, 1045, 3420 cm^−1: 1^H NMR (400 MHz, CDCl_3_): δ 2.57 (3H, s, N-CH_3_), 3.09–3.20 (4H, m, H-4, 5), 5.94, 6.09 (each 1H, d, *J* = 1.6 Hz, –OCH_2_O–), 6.56 (1H, s, H-3), 7.23–7.27 (3H, m, H-8, 9, 10), 8.07 (1H, d, *J* = 8.0 Hz, H-11); ESI-MS *m*/*z*:279 [M]^+^ (Figure 2).

**7-Hydroxydehydronuciferine** (**10**) as in [16], brown powder (MeOH); UV λ_max_: 254, 366 nm; IR ν_max_: 1625, 3430 cm^−1; 1^H NMR (400 MHz, CDCl_3_): δ 2.23 (3H, s, N-CH_3_), 3.28 (2H, m, H-4), 3.38 (2H, m, H-5), 3.89 (3H, s, C_1_–OCH_3_), 4.02 (3H, s, C_2_–OCH_3_), 6.65 (1H, br s, C_7_–OH), 7.03 (1H, s, H-3), 7.34 (1H, td, *J* = 8.4, 1.6 Hz, H-9), 7.45 (1H, td, *J* = 7.2, 1.6 Hz, H-10), 7.65 (1H, dd, *J* = 8.0, 1.2 Hz, H-8), 9.46 (1H, d, *J* = 8.4, H-11); ESI-MS *m*/*z*: 309 [M]^+^ (Figure 2).

**Cepharadione B** (**11**) as in [17], orange prisms (EtOAc); UV λ_max_: 213, 240, 301, 315, 440 nm; IR ν_max_: 1647, 1665 cm^−1; 1^H NMR (400 MHz, CDCl_3_): δ 3.90 (3H, s, N-CH_3_), 4.12, 4.15 (each 3H, s, OCH_3_–1, 2), 7.55 (1H, s, H-7), 7.67 (2H, m, H-9, 10), 7.90 (1H, m, H-8), 8.31 (1H, s, H-3), 9.55 (1H, m, H-11); ESI-MS *m*/*z*: 321 [M]^+^ (Figure 2).

### 2.2. Cestocidal Activity against *H. nana*

**Figure 3.**
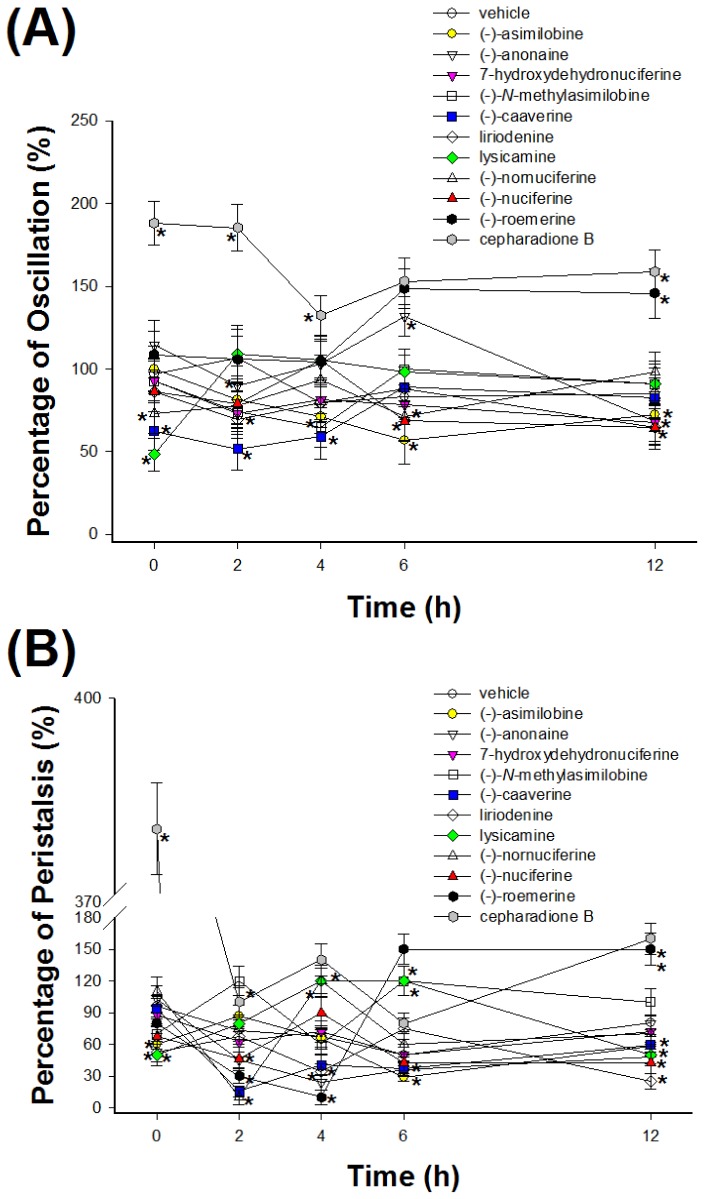
Effect of chemical constituents of *Nelumbo nucifera* Gaertn. cv. *Rosa-plena*. on *H. nana*. Treatment of liriodenine (**1**), lysicamine (**2**), (-)-anonaine (**3**), (-)-asimilobine (**4**), (-)-caaverine (**5**), (-)-*N*-methylasimilobine (**6**), (-)-nuciferine (**7**), (-)-nornuciferine (**8**), (-)-roemerine (**9**), 7-hydroxydehydronuciferine (**10**) and cepharadione B (**11**) (100 μM) with incubation times of 2, 4, 6 and 12 h on *H. nana*, respectively. Time course of effect on oscillation (**A**) and peristalsis (**B**) of *H. nana* on test compounds presented as percentages. Vehicle is 0.1% DMSO solvent. Each value is presented as mean ± SD of three individual experiments; * *p* < 0.05 indicates a significant difference from the result for vehicle-treated worms.

Figure 3a,b plot the time course of oscillation and peristalsis during liriodenine (**1**), lysicamine (**2**), (-)-anonaine (**3**), (-)-asimilobine (**4**), (-)-caaverine (**5**), (-)-*N*-methylasimilobine (**6**), (-)-nuciferine (**7**), (-)-nornuciferine (**8**), (-)-roemerine (**9**), 7-hydroxydehydronuciferine (**10**) and cepharadione B (**11**) treatment of 2, 4, 6 and 12 h. In oscillation activity assay, vehicle control (0.1% dimethyl sulphoxide (DMSO)) was decreased by 16% from 12 h cultivation (Figure 3a). However, in the peristalsis activity assay, vehicle control (0.1% DMSO) was decreased by 20% from 12 h cultivation (Figure 3b). The change of peristalsis of *H. nana* was more sensitive than oscillation via treatment of vehicle. Treatment with 100 μM liriodenine (**1**), lysicamine (**2**), (-)-anonaine (**3**), (-)-asimilobine (**4**), (-)-caaverine (**5**), (-)-*N*-methylasimilobine (**6**), (-)-nuciferine (**7**), (-)-nornuciferine (**8**), (-)-roemerine (**9**), 7-hydroxy dehydronuciferine (**10**) and cepharadione B (**11**) has a greater effect of peristalsis than on oscillation for 2, 4, 6 and 12 h.

**Figure 4.**
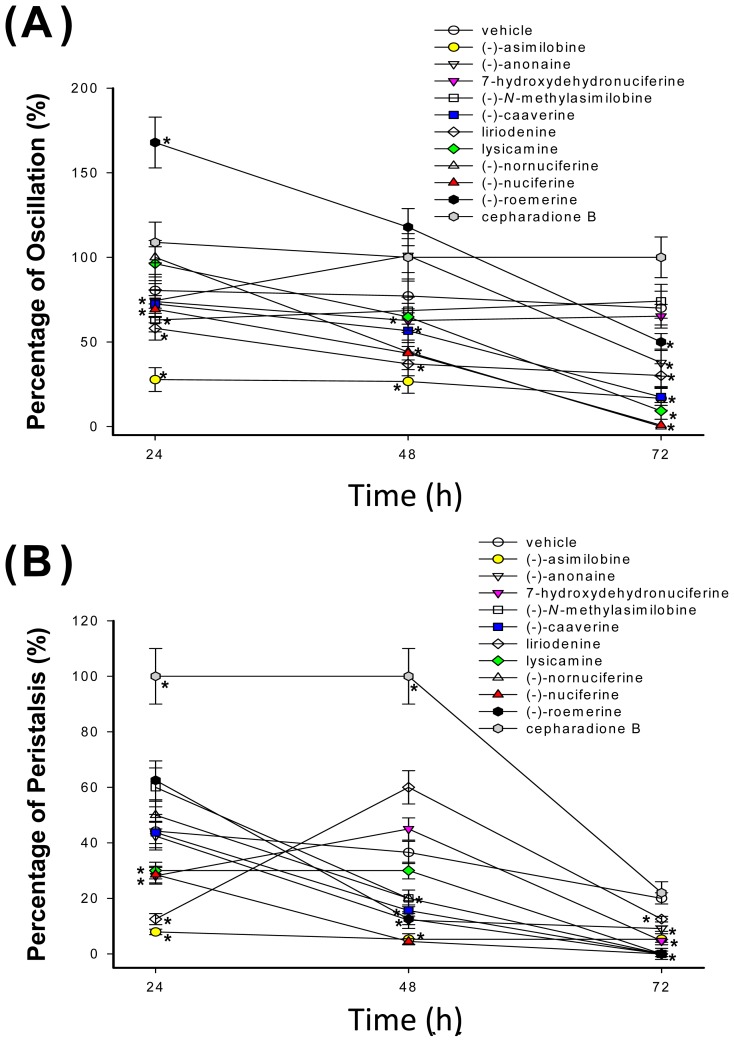
Effect of chemical constituents of *Nelumbo nucifera* Gaertn. cv. *Rosa-plena*. on *H. nana*. Treatment of liriodenine (**1**), lysicamine (**2**), (-)-anonaine (**3**), (-)-asimilobine (**4**), (-)-caaverine (**5**), (-)-*N*-methylasimilobine (**6**), (-)-nuciferine (**7**), (-)-nornuciferine (**8**), (-)-roemerine (**9**), 7-hydroxydehydronuciferine (**10**) and cepharadione B (**11**) (100 μM) with incubation times of 24, 48 and 72 h on *H. nana*, respectively. Time course of effect on oscillation (**A)** and peristalsis (**B**) of *H. nana* of test compounds presented as percentages. Vehicle is 0.1% DMSO solvent. Each value is presented as mean ± SD of three individual experiments; * *p* < 0.05 indicates a significant difference from the result for vehicle-treated worms.

Figure 4a,b plot the time course of oscillation and peristalsis during liriodenine (**1**), lysicamine (**2**), (-)-anonaine (**3**), (-)-asimilobine (**4**), (-)-caaverine (**5**), (-)-*N*-methylasimilobine (**6**), (-)-nuciferine (**7**), (-)-nornuciferine (**8**), (-)-roemerine (**9**), 7-hydroxydehydronuciferine (**10**) and cepharadione B (**11**) treatment of 24, 48 and 72 h. In oscillation and peristalsis activity assay, vehicle control (0.1% DMSO) was decreased to 70% and 20%, respectively from 24 to 72 h cultivation (Figure 4a,b).

In the oscillation activity assay (Figure 4a), exposure to 100 μM (-)-nornuciferine for 72 h caused the maximum cestocidal effect of *H. nana* without any oscillation activity. In addition, in the peristalsis activity assay (Figure 4b), exposure to 100 μM (-)-*N*-methylasimilobine, (-)-caaverine, lysicamine, (-)-nornuciferine, (-)-nuciferine and (-)-roemerine for 72 h cause the maximum cestocidal effect of *H. nana* without any peristalsis activity. This cestocidal effect on peristalsis is stronger than on oscillation activity. Peristalsis activity disappeared before oscillation activity was lost when *H. nana* died. This demonstrates that (-)-nornuciferine have maximum cestocidal effect than above compounds. The order of the cestocidal effect of the compounds was: (-)-nornuciferine > (-)-nuciferine > lysicamine > (-)-asimilobine ≥ (-)-caaverine > liriodenine ≥ (-)-anonaine > (-)-roemerine. However, 7-hydroxydehydronuciferine, (-)-*N*-methylasimilobine and cepharadione B have no cestocidal effect against *H. nana*.

### 2.3. Nematocidal Activity against *A. simplex*

In the first series of experiments, the larvicidal effects were used to study the ability of liriodenine (**1**), lysicamine (**2**), (-)-anonaine (**3**), (-)-asimilobine (**4**), (-)-caaverine (**5**), (-)-*N*-methylasimilobine (**6**), (-)-nuciferine (**7**), (-)-nornuciferine (**8**), (-)-roemerine (**9**), 7-hydroxydehydronuciferine (**10**) and cepharadione B (**11**) to alter survival of AsL3. In the time-dependent course shown above compounds have no nematocidal activity against *A. simplex* (AsL3). Additionally, the loss of spontaneous movement of AsL3 did not occur at a concentration of 100 μM of liriodenine (**1**), lysicamine (**2**), (-)-anonaine (**3**), (-)-asimilobine (**4**), (-)-caaverine (**5**), (-)-*N*-methylasimilobine (**6**), (-)-nuciferine (**7**), (-)-nornuciferine (**8**), (-)-roemerine (**9**), 7-hydroxydehydronuciferine (**10**) and cepharadione B (**11**). However, according assay of cestocidal activity against *H. nana* and nematocidal activity against *A. simplex*, we found above compounds shown more lethal efficacy of *H. nana* than against *A. simplex.*

### 2.4. Determination of Oxygen Radical Absorbing Capacity (ORAC)

**Figure 5.**
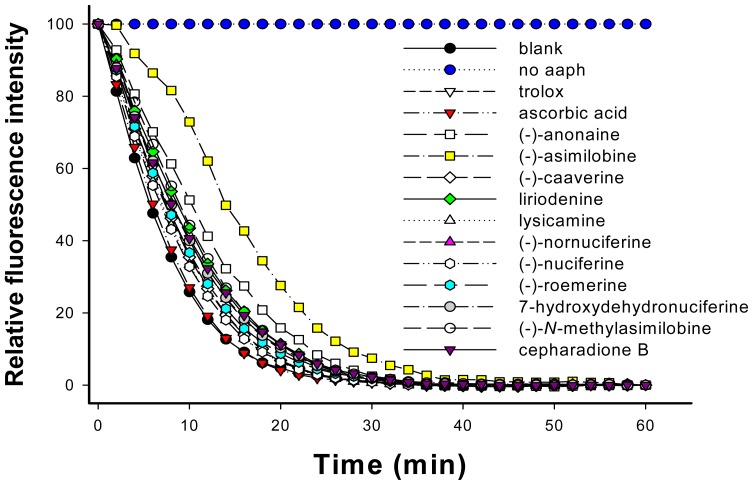
Time course of changes in fluorescence decay curve of fluorescein in the presence of 1 μM Trolox, ascorbic acid, liriodenine (**1**), lysicamine (**2**), (-)-anonaine (**3**), (-)-asimilobine (**4**), (-)-caaverine (**5**), (-)-*N*-methylasimilobine (**6**), (-)-nuciferine (**7**), (-)-nornuciferine (**8**), (-)-roemerine (**9**), 7-hydroxydehydronuciferine (**10**) and cepharadione B (**11**), respectively. For calculation of the area under the curve (AUC), please see Equation (2) in Section 3.7.

The peroxyl radical absorbing abilities of Trolox, ascorbic acid, liriodenine (**1**), lysicamine (**2**), (-)-anonaine (**3**), (-)-asimilobine (**4**), (-)-caaverine (**5**), (-)-*N*-methylasimilobine (**6**), (-)-nuciferine (**7**), (-)-nornuciferine (**8**), (-)-roemerine (**9**), 7-hydroxydehydronuciferine (**10**) and cepharadione B (**11**) as determined by oxygen radical absorbance capacity (ORAC) fluorescein assay are shown in Figure 5.

**Figure 6.**
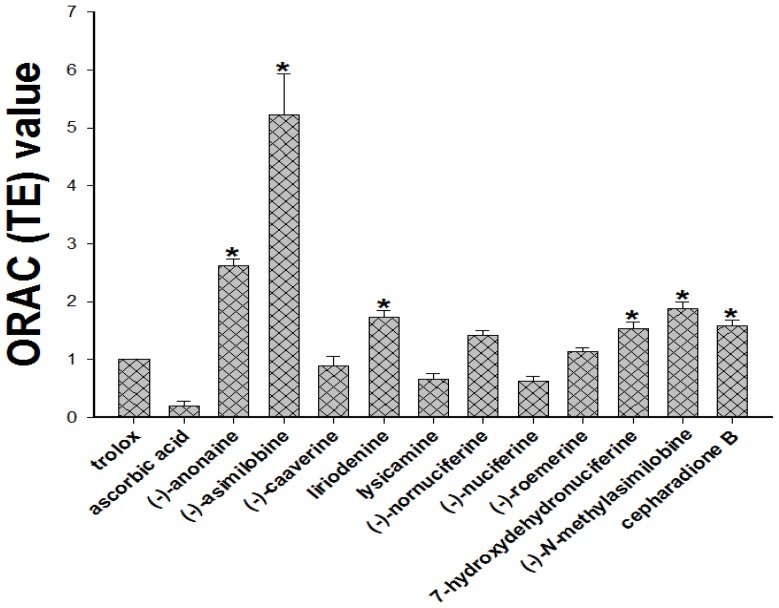
The ORAC (Trolox equivalents, TE) value was calculated by dividing the area under the sample curve by the area under the Trolox curve, with both areas being corrected by subtracting the area under the blank curve. Trolox and ascorbic acid were used as a positive control. The results represent the mean ± SD for three independent experiments. Statistically significant, * *p* < 0.05 to Trolox group.

When the test compounds were added, the relative fluorescence intensity decreased. Fluorescein was exposed to excitation light at 495 nm in the absence of AAPH over a 60-min period. There were no significant changes in fluorescence intensity over 60 min, suggesting that 0.06 μM fluorescein is photostable under such conditions. As shown in Figure 6, at a concentration of 1.0 μM, the average ORAC values of Trolox, ascorbic acid, liriodenine (**1**), lysicamine (**2**), (-)-anonaine (**3**), (-)-asimilobine (**4**), (-)-caaverine (**5**), (-)-*N*-methylasimilobine (**6**), (-)-nuciferine (**7**), (-)-nornuciferine (**8**), (-)-roemerine (**9**), 7-hydroxydehydronuciferine (**10**) and cepharadione B (**11**) for relative Trolox equivalents (TE) ORAC were determined to be 1.00, 0.20, 1.73, 0.67, 2.62, 5.23, 0.89, 1.89, 0.63, 1.43, 1.14, 1.54 and 1.59, respectively (Figure 6). The order of the ORAC values of the compounds was: (-)-asimilobine > (-)-anonaine > (-)-*N*-methylasimilobine > liriodenine > cepharadione B ≥ 7-hydroxydehydronuciferine > (-)-nornuciferine > (-)-roemerine > Trolox > (-)-caaverine > lysicamine ≥ (-)-nuciferine > ascorbic acid.

Thus those compounds have radical scavenging activity, not reduced cestocidal activity against *H. nana*. In ORAC fluorescein assay, (-)-asimilobine, (-)-anonaine, liriodenine, (-)-nornuciferine and (-)-roemerine were evaluated as radical scavengers compared to ascorbic acid and Trolox. A previous study demonstrated that not only is scavenging activity not involved in larvicide activity for *A. simplex*, but also that free radicals could harm the *A. simplex*; in this case, the scavenging of these free radicals allows for larvae survival [18]. However, in this study, (-)-asimilobine, (-)-anonaine, liriodenine, (-)-nornuciferine and (-)-roemerine not only have cestocidal activity for adult worms of *H. nana*, but also have inhibitory radical scavenging activity against peroxyl radical. This finding is consistent with *A. simplex* and *H. nana* [18]. Nevertheless, further investigations are necessary for the mode of aporphine actions and/or mechanisms for its cestocidal effects between free radical scavenging activity. Our results further demonstrated that (-)-asimilobine, (-)-anonaine and liriodenine have cestocidal activities against *H. nana* and reduced spontaneous movement of oscillation and peristalsis significantly and also have free radical scavenging activity that did not adversely affect their cestocidal activity. The above results might contribute to the search for more selective and efficient naturally cestocidal compounds.

## 3. Materials and Methods

### 3.1. Drugs and Chemicals

Dulbecco’s Modified Eagle Medium (DMEM), RPMI-1640, fetal bovine serum (FBS), l-glutamine, streptomycin, penicillin G, amphotericin B and all other cell culture reagents were obtained from Gibco BRL Life Technologies (Grand Island, NY, USA). Fluorescein, 6-hydroxy- 2,5,7,8-tetramethylchroman-2-carboxylic acid (Trolox) and 2,2′-Azobis(2-amidinopropane) dihydrochloride (AAPH) were obtained from Sigma-Aldrich Chemical Co. (St. Louis, MO, USA). All drugs and reagents were dissolved in sterilized distilled H_2_O unless otherwise noted. Liriodenine (**1**), lysicamine (**2**), (-)-anonaine (**3**), (-)-asimilobine (**4**), (-)-caaverine (**5**), (-)-*N*-methylasimilobine (**6**), (-)-nuciferine (**7**), (-)-nornuciferine (**8**), (-)-roemerine (**9**), 7-hydroxydehydronuciferine (**10**) and cepharadione B (**11**) were dissolved in DMSO at 1 M stock and serially diluted with sterilized distilled H_2_O and vehicle (contains 1% DMSO in sterilized distilled H_2_O).

### 3.2. Extraction and Isolation

The leaves of *Nelumbo nucifera* Gaertn. cv. *Rosa-plena* were collected from Tainan, Taiwan, November 2008. Plant material was identified by Dr. Fu-Yuan Lu (Department of Forestry and Natural Resources College of Agriculture, National Chiayi University, Chiayi, Taiwan). A voucher specimen (*N. nucifera* Gaertn. cv. *Rosa-plena*) was deposited in the School of Medical and Health Sciences, Fooyin University, Kaohsiung, Taiwan. The air-dried leaves of *N. nucifera* Gaertn. cv. *Rosa-plena* (1.5 kg) were extracted with MeOH (50 L × 5) at room temperature and a MeOH extract (108.7 g) was obtained upon concentration under reduced pressure. The MeOH extract, suspended in H_2_O (1 L), was partitioned with CHCl_3_ (3 L × 4) to give fractions soluble in CHCl_3_ (57.2 g) and H_2_O (43.6 g). The CHCl_3_-soluble fraction was chromatographed over silica gel (1700 g, 70–230 mesh) using *n*-hexane/EtOAc/MeOH mixtures as eluents to produce six fractions. Part of fraction 3 (7.83 g) was subjected to silica gel chromatography, by eluting with *n*-hexane-acetone (7:1), enriched gradually with acetone, to furnish two fractions (3-1–3-2). Fraction 3-2 (2.13 g) was further purified on a silica gel column using *n*-hexane/acetone mixtures to obtain (-)-caaverine (**5**) (4 mg). Part of fraction 4 (10.27 g) was subjected to silica gel chromatography by eluting with *n*-hexane-acetone (5:1), enriched with acetone to furnish three further fractions (4-1–4-3). Fraction 4-1 (4.37 g) was further purified on a silica gel column using *n*-hexane-acetone mixtures to obtain lysicamine (**2**) (15 mg), 7-hydroxydehydronuciferine (**10**) (12 mg) and (-)-nornuciferine (**8**) (17 mg). Fraction 4-2 (3.05 g) was further purified on a silica gel column using *n*-hexane-acetone mixtures to obtain (-)-roemerine (**9**) (6 mg), (-)-nuciferine (**7**) (20 mg), (-)-anonaine (**3**) (5 mg) and cepharadione B (**11**) (15 mg). Fraction 4-3 (2.51 g) was further purified on a silica gel column using *n*-hexane-acetone mixtures to obtain (-)-asimilobine (**4**) (4 mg) and (-)-*N*-methylasimilobine (**6**) (16 mg). Part of fraction 5 (5.34 g) was subjected to silica gel chromatography by eluting with CH_2_Cl_2_/MeOH (40:1), enriched with MeOH to furnish two fractions (5-1–5-2). Fraction 5-1 (2.53 g) eluted with CH_2_Cl_2_/MeOH (30:1), was further separated using silica gel column chromatography and preparative TLC [CH_2_Cl_2_/MeOH (40:1)] and gave liriodenine (**1**) (4 mg)

### 3.3. Preparation of *H. nana* Adult Worms

*H. nana* adult worms were obtained from each part of the intestines of wild type mice, purchased from Lin’s farm in Fengshan, Kaohsiung, Taiwan. These parts of the duodenum, jejunum, ileum, colon and rectum were used. The *H. nana* adult worms ranged in length from 5–50 mm, and were collected using a needle with a blunt tip, before being placed in Petri dishes (Gibco BRL Life Technologies, Grand Island, NY, USA) with 0.9% NaCl and gentamycin (10 mg/mL). They were then washed several times. The adult worms were individually observed under an inverted microscope and those that exhibited any kind of internal or external damage were discarded. The adult worms were then identified by their morphological features, divided into groups and placed in 24-well plates contained cultivated media RPMI-1640 (Gibco BRL Life Technologies) plus 20% FBS, pH 7.4, in an atmosphere of 95% O_2_/5% CO_2_, 37 °C. These culture conditions have been shown to maximize the development and survival of *H. nana* [18].

### 3.4. Assay of Cestocidal Activity of Oscillation and Peristalsis Test on *H. nana*

The above *H. nana* cultivated media were supplemented with l-glutamine (2 mM), penicillin (100 U/mL), streptomycin (0.1 mg/mL) and amphotericin B (0.25 μg/mL), and then the effects of liriodenine (**1**), lysicamine (**2**), (-)-anonaine (**3**), (-)-asimilobine (**4**), (-)-caaverine (**5**), (-)-*N*-methylasimilobine (**6**), (-)-nuciferine (**7**), (-)-nornuciferine (**8**), (-)-roemerine (**9**), 7-hydroxy dehydronuciferine (**10**) and cepharadione B (**11**) at concentrations of 100 μM were tested. The survival and mobility of the adult worm were assessed at 2, 4, 6, 12, 24, 48, and 72 h using a stereomicroscope. They were observed for their spontaneous motility and evoked responses at 2, 4, 6, 12, 24, 48, and 72 h using a stereomicroscope. The oscillation and peristaltic states of adult worms were scored blindly by two investigators. Cestode activity was scored by monitoring both oscillation and peristalsis. Oscillation was scored of movement at scolex and neck for each second for 30 s, and then the highest score was 30. Peristalsis was recorded as the contraction real times at scolex and neck. All data were compared with the initial time before the test compounds had been added. Worm death and complete standstill were identified by no oscillation and peristaltic changes for 30 s. The mortality was recorded after ascertaining that the worms neither moved when shaken vigorously nor when dipped in warm medium [18,19].

### 3.5. *A. simplex* Larvae Preparation

The AsL3 were obtained from the muscle and peritoneum of fresh *Trichiurus lepturus* (largehead hairtail, Pacific cutlassfish) that were purchased from the fish market of Kaohsiung, Taiwan. The AsL3 had an average length of 20–22 mm, and were collected using a needle with a blunt tip, placed in Petri dishes with 0.9% NaCl and washed several times. The majority of the larvae were encysted, but they quickly became excysted upon washing in NaCl solution. They were individually observed under an inverted microscope and those that showed any internal or external damage were discarded. The larvae were then identified by morphological features, divided into groups and placed in 24-well plates containing cultivated media RPMI-1640 (Gibco BRL Life Technologies) plus 20% FBS, pH 4.0, in an atmosphere of 95% O_2_/5% CO_2_, 37 °C. These culture conditions have been shown to maximize the development and survival of *A. simplex* [19–21].

### 3.6. Assay of Nematocidal Activity on *A. simplex*

The above AsL3 cultivated media were supplemented with L-glutamine (2 mM), penicillin (100 U/mL), streptomycin (0.1 mg/mL) and amphotericin B (0.25 μg/mL), and tested of liriodenine (**1**), lysicamine (**2**), (-)-anonaine (**3**), (-)-asimilobine (**4**), (-)-caaverine (**5**), (-)-*N*-methylasimilobine (**6**), (-)-nuciferine (**7**), (-)-nornuciferine (**8**), (-)-roemerine (**9**), 7-hydroxydehydronuciferine (**10**) and cepharadione B (**11**) for 100 μM. The survival and mobility of the larvae were assessed at 2, 4, 8, 12, 24, 48 and 72 h using a stereomicroscope. Two investigators blindly scored the larvae as dead, with poor mobility or with normal mobility. The percentage losses of spontaneous motion during 3 min periods immediately after incubation and complete standstill were determined by stimulation 4–5 h later (defined as death). The mortality was recorded after ascertaining that the worms neither moved when shaken vigorously nor when dipped in warm medium. The nematocidal activity was modified according to a scoring system that was developed by Lin *et al.* [20].

### 3.7. Evaluation of Oxygen Radical Absorbing Capacity (ORAC)

The automated ORAC assay was carried out on a FLUOstar Galaxy plate reader (Roche Diagnostic System Inc., Branchburg, NJ, USA) as described in a previous report by Gillespie and co-workers [22]. The experiment was conducted at 37 °C under pH 7.0 condition with a blank sample in parallel. Briefly, AAPH was used as a peroxyl generator, and Trolox (1 μM), a water-soluble analogue of vitamin E, was used as a control standard. The final reaction mixture for each black microplate in a 96-well microplate assay contained fluorescent (0.06 μM), AAPH (18.75 mM) and appropriate test substance (1 μM) in phosphate buffer (75 mM). The analyzer was programmed to record the fluorescence of FL (FLUOstar Galaxy plate reader, Vienna, VA, USA) every minute after the addition of AAPH. All fluorescent measurements are expressed relative to the initial reading (excitation/emission at 495/530 nm). Parameters of assay for the plate reader were as follows: cycle number, 50; cycle time, 60 s; shaking time, 8 s before each cycle; and position delay, 0.5 s. Final ORAC values were calculated using the regression equation between Trolox concentration and the net area under curve (AUC) and are expressed as micromole Trolox equivalents per liter. The AUC was calculated as:

(1) $$AUC=60+f_{1}/f_{0}+\ldots f_{i}/f_{0}+\ldots +f_{59}/f_{0}+60(f_{60}/f_{0})$$

where *f*_0_ *=* initial fluorescence reading at 0 min and *fi =* fluorescence reading at time *i*. The data were analyzed using a Microsoft Excel macro program using this Equation (1) to calculate the AUC. The net AUC is obtained by subtracting the AUC of the blank from that of a sample. The relative Trolox equivalent ORAC value is calculated as:

(2) $$\begin{array}{c} \text{Relative }ORAC\;\text{value}=[({AUC}_{\text{sample}}-{AUC}_{\text{blank}})/({AUC}_{\text{Trolox}}-{AUC}_{\text{blank}})]\times \\ \left(\text{molarity of Trolox}/\text{molarity of sample}\right) \end{array}$$

### 3.8. Statistical Analysis

The results are expressed as mean ± standard deviation (SD). Statistical differences were estimated by one-way analysis of variance (ANOVA) followed by Dunnett’s test or the Tukey-Kramer test. A *p* value of 0.05 was regarded as significant. The data were analyzed and the figures plotted using software (SigmaPlot Version 8.0 and SigmaStat Version 2.03, Chicago, IL, USA).

## 4. Conclusions

This study is the first to determine the anthelmintic activities of *Nelumbo nucifera* Gaertn. cv. *Rosa-plena* against *A. simplex* and *H. nana.* Thus we found that oxoaporphines [liriodenine (**1**) and lysicamine (**2**)] and aporphines [(-)-anonaine (**3**), (-)-asimilobine (**4**), (-)-caaverine (**5**), (-)-nuciferine (**7**), (-)-nornuciferine (**8**), and (-)-roemerine (**9**)] have not only cestocidal activity for *H. nana* but also radical scavenging activity against peroxyl radical, respectively. So those compounds have radical scavenging activity that did not reduce their cestocidal activity against *H. nana*. Further investigations of the mode of *Nelumbo nucifera* Gaertn. cv. *Rosa-plena* constituents’s actions and/or mechanisms for its cestocidal effects between free radical scavenging activity are necessary. In addition, we found dehydroaporphines [7-hydroxydehydronuciferine (**10**) and cepharadione B (**11**)] have antioxidant capacity but no cestocidal and nematocidal effect against *H. nana* and *A. simplex*, respectively. These results might be useful in the search for more selective and efficient naturally anthelmintic compounds.
